# Relationship between Syringe Flow Measurements and Viscosity of Nectar-Thick Beverages for Dysphagia Management

**DOI:** 10.3390/foods10091981

**Published:** 2021-08-25

**Authors:** Yulim Jeong, Woobin Lim, Byoungseung Yoo

**Affiliations:** Department of Food Science and Biotechnology, Dongguk University-Seoul, Goyang 410-820, Korea; yulim1ah@dongguk.edu (Y.J.); dladnqls1234@naver.com (W.L.)

**Keywords:** dysphagia, thickened beverage, viscosity, syringe flow test, IDDSI

## Abstract

Commercial cold beverages thickened with a xanthan gum (XG)-based food thickener were examined at different thickness levels by using the simple, cost-effective syringe flow test (SFT) developed by the International Dysphagia Diet Standardization Initiative (IDDSI). We prepared cold thickened beverage (CTB) samples with different thickener concentrations and measured them by extrapolating the viscosity range (51–350 mPa·s) for nectar-like consistency. CTBs were also measured via the line-spread test (LST), and the flow distance value (cm) by LST and the volume remaining (mL) in the syringe by SFT was correlated with the apparent viscosity (*η_a,_*_50_). Plots comparing *η_a,_*_50_ with SFT or LST values showed good exponential relationships between the measurements. The SFT showed a better relationship (*R*^2^ = 0.928) than LST (*R*^2^ = 0.825), indicating that the former can predict the viscosity better in the range for nectar-like consistency. In particular, the SFT showed a significant difference (*R*^2^ = 0.964) compared to the LST (*R*^2^ = 0.709) for thickened protein-based beverages. These results suggest that the SFT using the IDDSI methodology is a more suitable instrument than the LST for accurately evaluating the viscosity of XG-based CTBs with nectar-like consistency.

## 1. Introduction

Oropharyngeal dysphagia refers to swallowing problems when transporting solid or liquid food from the mouth to the esophagus that can lead to aspiration pneumonia [1,2]. Therefore, liquid foodstuffs are commonly thickened with commercial thickener powder to modify their viscosity [3,4]. The effective treatment of dysphagia requires pertinent viscosity-modification of thickened liquids because inaccuracies in preparing them at the targeted viscosity can cause aspiration [5,6]. Therefore, the use of a sophisticated viscometer or rheometer is needed to provide thickened liquids with more precise viscosity or consistency [7]. However, these instruments are impractical because they are expensive and inconvenient to use in most clinical care settings. Therefore, the line-spread test (LST) or the syringe flow test (SFT) introduced by the International Dysphagia Diet Standardization Initiative (IDDSI) has been used as an empirical method in hospitals for estimating the thickness level or viscosity of thickened liquids because the LST and SFT are inexpensive, practical, and simple clinical tools [7,8,9,10,11,12,13]. In general, the IDDSI levels for thickened fluids are classified into five different levels (0–4) established by the SFT as a practical measurement of flow: level 0 (thin), level 1 (slightly thick), level 2 (mildly thick), level 3 (moderately thick), and level 4 (extremely thick) [14]. However, it is known that there is no correlation between the viscosity classification regimes provided by the National Dysphagia Diet Task Force (NDDTF) and IDDSI [15].

The LST method provides the means to predict the viscosity of thickened fruit juices prepared with different food thickeners at different concentrations that shows a strong exponential relationship between rheometer-measured viscosity and LST values [7,8]. In addition, Kim et al. [9] investigated the applicability of the SFT by examining the relationship between the apparent viscosity (*η_a,_*_50_) by using a rheometer and the remaining liquid volume in the syringe after SFT or the line-spread distance after LST for water thickened with xanthan gum (XG)-based or starch-based food thickeners. They found that there is a better relationship between the LST and *η_a,_*_50_ values than with SFT at three different thickness levels (nectar-like (51–350 mPa·s), honey-like (351–1750 mPa·s), and pudding-like (>1750 mPa·s)) established by the NDDTF, suggesting that the SFT is not suitable for measuring the practical viscosity of liquids thickened with starch-based food thickeners. They also concluded that the SFT may be suitable for measuring only XG-based thickened liquids with low viscosity (such as nectar-thick liquids) because very little or no liquid flows from the syringe at higher thickener concentrations (>1.0%) due to the smaller size of the syringe opening compared to the sample cylinder size for the LST. Recently, Cote et al. [11] suggested that a wider variety of beverages is needed for a study comparing SFT or LST values with rheometer parameters because these instruments can be greatly influenced by the composition of the beverages. Although much research has been done on the SFT by IDDSI, no attempt has been made to explore the relationship between *η_a,_*_50_ and the SFT values for various cold thickened beverage (CTB) samples with low viscosity, such as nectar-thick liquids which are in the viscosity range of 51–350 mPa·s.

Our aim was to study the reliability of SFT by investigating the relationship between the apparent viscosity and the amount of remaining sample after the SFT for various nectar-like CTBs prepared with an XG-based thickener at a concentration of less than 1.0%. In addition, the SFT was compared with the commonly used LST toward providing a reliable instrument for accurately estimating the viscosity of CTBs. Selecting an appropriate method for measuring the viscosity of nectar-thick liquids will practically assist clinicians or care helpers to prepare CTBs with the desired thickness level for safe swallowing.

## 2. Materials and Methods

### 2.1. Materials and Sample Preparation

The dispersing media used were seven commercially available beverages marketed in Korea, including cold beverages such as orange juice (Coca-Cola Beverage Co., Yangsan, Korea), grape juice (Coca-cola Beverage Co., Yangsan, Korea), apple juice (Woongjin Foods Co. Ltd., Kongju, Korea), sports drink (Donga-Otsuka, Co., Ltd., Cheongju, Korea), whole milk (Seoul Milk, Co. Ltd., Seoul, Korea), low-fat milk (Seoul Milk, Co., Ltd., Seoul, Korea), and soybean milk (Dr. Chung’s Food Co. Ltd., Cheongju, Korea). All food beverages were bought at a local market by selecting products with an equivalent sell-by date. The nutritional information of the beverage products are shown in Table 1. All dispersing media were thickened with a commercial XG-based food thickener powder (composed of XG, guar gum, and dextrin) (Visco-up, Rheosfood Inc., Seoul, Korea). CTB samples were prepared by mixing the thickener powder with the corresponding beverage at 8 °C while softly stirring for 60 s, and then stored in a refrigerator at 4 °C for 1 h before being used in the experiments. We investigated several concentration ranges (0.2–1.0%, *w*/*w*) of thickener for each beverage and measured them by extrapolating only the nectar-thick liquids.

### 2.2. Viscosity Measurements

The flow rheological properties of the CTBs were measured with a rheometer (Rheostress1, Haake GmbH, Karlsruhe, Germany) using plate-plate geometry (35 mm diameter and 500 µm gap). Each sample was applied between the parallel plates at 8 °C. The flow properties were evaluated after 5 min stabilization at 8 ± 0.1 °C. Experimental shear stress vs. shear rate was measured over the shear rate bounds of 0.1–100 s^−1^ by using the power-law model.

(1) $$\sigma =K\cdot {\dot{\gamma }}^{n}$$

where $\dot{\gamma }$ is the shear rate (s^−1^), σ is the shear stress (Pa), *K* is the consistency index (Pa·s^n^), and *n* is the flow behavior index. The apparent viscosity *(η_a,_*_50_) was calculated at 50 s^−1^ after obtaining the values of *K* and *n*.

### 2.3. Syringe Flow Test (SFT)

**Figure 1:**
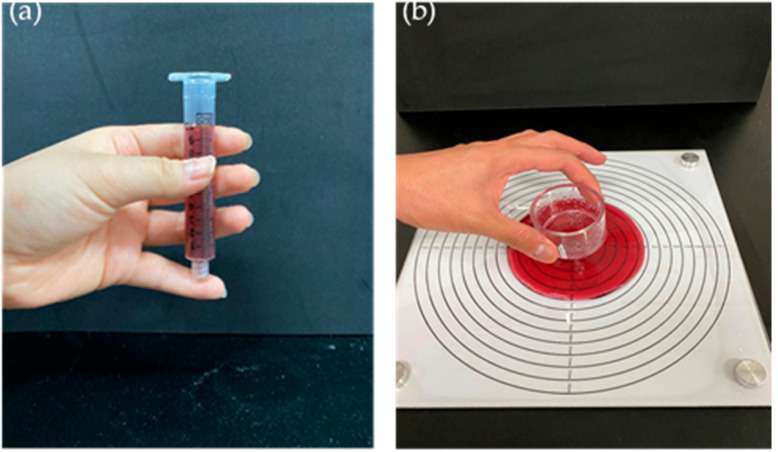
(**a**) syringe flow test (SFT) and (**b**) line-spread test (LST) for thickened beverages.

A reference 10 mL Luer-Lok tip syringe (Becton Dickinson Medical Pte., Ltd., Singapore) was used for the SFT of the CTBs. A 10 mL sample was applied in the syringe with a blocked nozzle and then released with the fingers at room temperature for 10 s (Figure 1a). The remaining liquid volume in the syringe was measured to obtain the result of the SFT.

### 2.4. Line Spread Test (LST)

The LST was used to calculate the spread distance of the CTBs (Figure 1b). A cylindrical tube (height: 3.5 cm, diameter: 5.0 cm) filled with the CTB was loaded on a plastic board labeled with concentric circles and allowed to stabilize at room temperature for 5 min. The tube was then lifted, and the CTB sample was allowed to expand outward for 60 s. The expanded distance was the average of the readings for the four quadrants of the spread.

### 2.5. Statistical Analysis

All experiments were measured three times and each experimental data item is reported as the mean and standard deviation. Duncan’s multiple range test based on analysis of variance (ANOVA) was used to reveal statistically significant differences among the mean values (*p* < 0.05). Regression analyses were carried out to determine whether the relationships between the *η_a,_*_50_ values and SFT or LST measurements of the CTB samples were significant.

## 3. Results and Discussion

### 3.1. Determination of Nectar-Thick Cold Thickened Beverage (CTB) Samples from Viscosity Measurements

*η_a,_*_50_ values of all CTB samples in the thickener concentration range of 0.2–1.0% were measured to determine the thickener concentrations within the viscosity range for the nectar-thick liquid, and the *η_a,_*_50_ results are reported in Table 2. In general, significant differences in *η_a,_*_50_ values were found for the CTBs prepared with different thickener concentrations (*p* < 0.05), suggesting that the *η_a,_*_50_ value is greatly dependent on the thickness level. Meanwhile, the *η_a,_*_50_ values of all of the CTBs were in the range of 0.02–0.46 Pa·s for three of the “consistency” classes established by the NDDTF: thin-like (<50 mPa·s), nectar-like (51–350 mPa·s), and honey-like (351–1750 mPa·s). The *η_a,_*_50_ values of each CTB significantly increased with an increase in thickener concentration, indicating that small differences in the amount of XG-based thickener produced significant differences in viscosity (*p* < 0.05). Moreover, the CTB samples had different *η_a,_*_50_ values for the same thickener concentration.

The CTBs prepared with whole milk and low-fat milk showed much higher *η_a,_*_50_ values than the others. In particular, when comparing the *η_a,_*_50_ values of the CTBs, the thickened whole milk and low-fat milk samples showed much higher viscosity values as honey-like liquids at concentrations of more than 0.8% than the others, and there were also greater differences between the thickener concentrations used. The differences in *η_a,_*_50_ values of the CTBs can be attributed to the type of beverage as a dispersing medium, as suggested by Cho and Yoo [2] and Kim and Yoo [16]. They explained that the pronounced *η_a,_*_50_ values of CTBs prepared with whole milk and low-fat milk can be interpreted as the result of interaction between the various constituents (protein, fat, and carbohydrate) in the milk and XG in the thickener. It has also been reported that protein molecules entangle the water molecules interacting with XG, thereby increasing the viscosity of a thickened protein-based beverage [17].

Based on the *η_a,_*_50_ values of the CTBs, it was found that the thickener concentrations for the nectar-thick viscosity range (51–350 mPa·s) of all CTB samples were in the range of 0.3–1.0% (orange juice: 0.4–0.9%; grape juice: 0.4–1.0%; apple juice: 0.3–1.0%; the sports drink: 0.3–1.0%; whole milk: 0.3–0.8%; low-fat milk: 0.3–0.8%; and soybean milk: 0.3–0.9%). CTBs within the nectar-thick viscosity range also yielded three thickness categories (slightly thick (level 1), mildly thick (level 2), and moderately thick (level 3)) established via the IDDSI syringe flow test, as reported in Table 2. Consequently, we measured the thickness levels of the selected CTB samples within the nectar-thick viscosity range using the LST and SFT methods.

### 3.2. SFT and LST Measurements within the Nectar-Thick Viscosity Range

Table 3 summarizes the SFT and LST measurements of the CTB samples within the nectar-thick viscosity range (51–350 mPa·s). In the case of the SFT method, the remaining volume values of CTB samples within the nectar-thick viscosity range were 2.73–9.87 mL, indicating that they yielded three levels of thickness (level 1: remaining volume of 1–4 mL; (level 2: remaining volume of 4–8 mL; and level 3: remaining volume of 8–10 mL), as measured via the SFT method. In general, significant differences in the SFT and LST values were found for CTBs prepared at different thickener concentrations (*p* < 0.05). This indicates that small differences in the thickener concentration produced significant changes in the flow values measured via SFT and LST. In particular, to evaluate between the thickener concentrations used, the range in volume values measured via SFT (2.73–9.87 mL) was greater than for the distance values measured via LST (5.73–9.93 cm). These findings suggest that a volume measurement method is better for differentiating between thickness levels of various CTBs prepared with different concentrations (0.3–1.0%) of XG-based thickener within the nectar-thick viscosity range.

### 3.3. Relationship between η_a,50_ Values and SFT or LST Values

**Figure 2:**
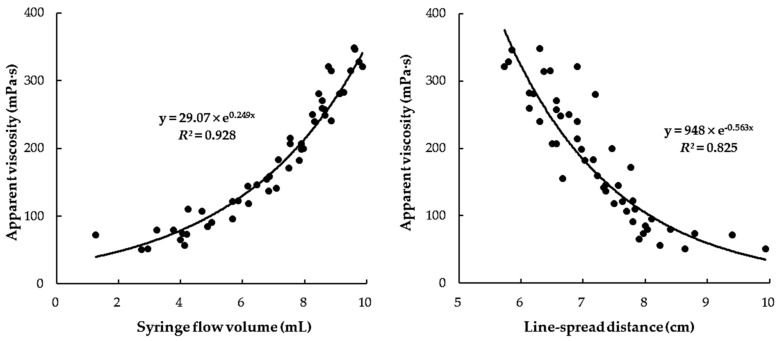
Correlation between apparent viscosity (*η_a,_*_50_) and flow volume (mL) by SFT or flow distance (cm) by LST of all thickened beverages within the nectar-thick viscosity range (51–350 mPa·s).

Figure 2 shows the relationship between the *η_a,_*_50_ values measured with a rheometer and the volume or distance values measured via the SFT and LST methods, respectively. The SFT volume values increased with increasing *η_a,_*_50_ value, whereas the LST distance values increased with decreasing *η_a,_*_50_ value. Plots of the *η_a,_*_50_ values vs. SFT or LST values revealed exponential relationships, which is in good agreement with previous studies of thickened beverages with higher thickener concentrations (>1.0%) [7,9]. In addition, *η_a,_*_50_ values vs. the SFT values revealed a better exponential relationship (*R*^2^ = 0.928) than that of *η_a,_*_50_ values vs. LST values (*R*^2^ = 0.825). This result indicates that the SFT is suitable for measuring the practical viscosity of CTBs with an XG-based thickener, which contradicts the study of Kim et al. [7] that showed a stronger relationship between LST and *η_a,_*_50_ values than SFT and *η_a,_*_50_ values for thickened water samples prepared with higher thickener concentrations (1.0–3.0%). According to the authors, the relationship between SFT and *η_a,_*_50_ values becomes poor when a thickened liquid sample prepared with a higher thickener concentration cannot flow out of the syringe. Therefore, they proposed that the ability of SFT to determine the thickness levels of CTBs with high viscosity is limited. From these results, it was found that differentiating the thickness levels with SFT is suitable for CTBs in the nectar-thick viscosity range (51–350 mPa·s).

**Figure 3:**
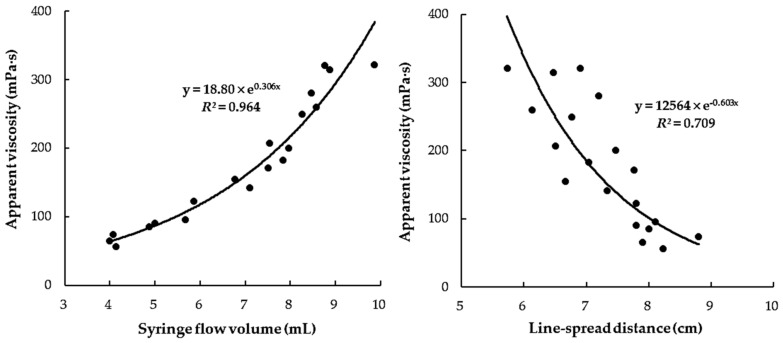
Correlation between apparent viscosity (*η_a,_*_50_) and flow volume (mL) by SFT or flow distance (cm) by LST of protein-based thickened beverages in nectar-thick range (51–350 mPa·s).

**Figure 4:**
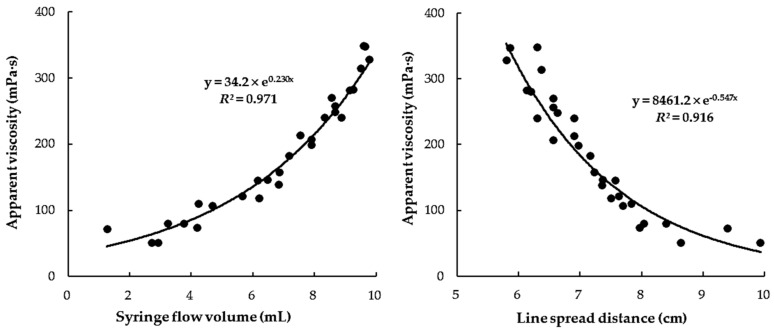
Correlation between apparent viscosity (*η_a,_*_50_) and flow volume (mL) by SFT or flow distance (cm) by LST values of fruit juice and sports drink-based thickened beverages in nectar-thick range (51–350 mPa·s).

Figure 3 shows the relationship between the *η_a,_*_50_ and LST or SFT values for the protein-based CTBs (whole milk, low-fat milk, and soybean milk) only. It can be seen that the relationship (*R*^2^ = 0.709) between *η_a,_*_50_ and LST values was much weaker than that (*R*^2^ = 0.964) between *η_a,_*_50_ and SFT values. This result suggests that flow measurements via the LST method may be influenced by the changed rheological characteristics (such as the viscoelastic property) due to strong interaction between the protein components in beverages and XG in the food thickener [11,16]. However, the LST and SFT methods both showed much better relationships for the fruit juice and sports drink-based CTBs (*R*^2^ = 0.971 for SFT; *R*^2^ = 0.916 for LST) (Figure 4) compared with the relationships for the protein-based CTBs (*R*^2^ = 0.964 for SFT; *R*^2^ = 0.709 for LST). From these observations, it can be concluded that the relationship between the viscosity and LST of all of the CTBs is greatly influenced by the constituents in the CTB, and so the LST is not suitable for differentiating the thickness levels of protein-based CTBs. In addition, these results suggest that the SFT method is a simple and reliable clinical measurement tool for healthcare providers, speech therapists, and patients who need to prepare CTBs with a targeted level of nectar-like thickness.

## 4. Conclusions

For various CTB samples prepared with an XG-based thickener within the nectar-thick viscosity range (51–350 mPa·s), the differences in volume values between thickener concentrations measured via the SFT were greater than those by using distance values measured via LST. There were better relationships between the *η_a,_*_50_ values measured with a rheometer and the flow volume (mL) values via the SFT compared to via the LST. In particular, for the protein-based CTBs, the relationship between *η_a,_*_50_ and SFT values was much stronger than that between *η_a,_*_50_ and LST values. Therefore, the SFT is a better method than the LST for predicting the viscosity of nectar-thick CTBs. The findings of this study reveal that the SFT is a simple and reliable clinical measurement tool to assess the thickness levels of XG-based thickened liquids within a low viscosity range. For accurately determining correct thickness levels of thickened liquids, specific information (such as the thickener concentration and the type of liquid) is required before the SFT can be conducted sugar binder solution in the FBA process.

## Figures and Tables

**Table 1:** Composition for the beverages used in this study.

Type of Beverage	Nutrition (per 100 mL)
Orange juice	Protein (<0.5 g), carbohydrate (12 g), fat (<0.5 g)
Grape juice	Protein (<0.5 g), carbohydrate (14 g), fat (<0.5 g)
Apple juice	Protein (<0.5 g), carbohydrate (11.1 g), fat (<0.5 g)
Sports drink	Protein (<0.5 g), carbohydrate (6.0 g), fat (<0.5 g)
Whole milk	Protein (3.0 g), carbohydrate (4.5 g), fat (3.6 g)
Low fat milk	Protein (3.0 g), carbohydrate (4.5 g), fat (1.0 g)
Soybean milk	Protein (3.2 g), carbohydrate (4.2 g), fat (3.7 g)

**Table 2:** Apparent viscosity (*η_a,_*_50_, Pa·s) values of cold thickened beverages with different thickener concentrations.

Thickener Concentration (%)	Apparent Viscosity (*η_a,_*_50_, Pa·s)						
	Orange Juice	Grape Juice	Apple Juice	Sports Drink	Whole Milk	Low Fat Milk	Soybean Milk
0.2	0.02 ± 0.00 ^i^	0.02 ± 0.00 ^i^	0.03 ± 0.00 ^i^	0.03 ± 0.00 ^i^	0.04 ± 0.00 ^i^	0.04 ± 0.00 ^i^	0.04 ± 0.00 ^i^
0.3	0.04 ± 0.00 ^h^	0.04 ± 0.00 ^h^	0.05 ± 0.00 ^h^	0.05 ± 0.00 ^h^	0.07 ± 0.00 ^h^	0.07 ± 0.00 ^h^	0.06 ± 0.00 ^h^
0.4	0.08 ± 0.00 ^g^	0.07 ± 0.00 ^g^	0.08 ± 0.00 ^g^	0.07 ± 0.00 ^g^	0.10 ± 0.00 ^g^	0.09 ± 0.00 ^g^	0.08 ± 0.00 ^g^
0.5	0.12 ± 0.00 ^f^	0.11 ± 0.00 ^f^	0.12 ± 0.00 ^f^	0.11 ± 0.00 ^f^	0.17 ± 0.01 ^f^	0.14 ± 0.01 ^f^	0.12 ± 0.00 ^f^
0.6	0.16 ± 0.00 ^e^	0.14 ± 0.00 ^e^	0.15 ± 0.00 ^e^	0.14 ± 0.00 ^e^	0.20 ± 0.01 ^e^	0.18 ± 0.00 ^e^	0.15 ± 0.00 ^e^
0.7	0.20 ± 0.00 ^d^	0.18 ± 0.00 ^d^	0.21 ± 0.00 ^d^	0.21 ± 0.01 ^d^	0.28 ± 0.00 ^d^	0.25 ± 0.00 ^d^	0.21 ± 0.00 ^d^
0.8	0.25 ± 0.01 ^c^	0.24 ± 0.00 ^c^	0.27 ± 0.00 ^c^	0.24 ± 0.00 ^c^	0.32 ± 0.00 ^c^	0.31 ± 0.00 ^c^	0.26 ± 0.00 ^c^
0.9	0.31 ± 0.00 ^b^	0.26 ± 0.00 ^b^	0.28 ± 0.01 ^b^	0.28 ± 0.00 ^b^	0.44 ± 0.00 ^b^	0.41 ± 0.00 ^b^	0.32 ± 0.00 ^b^
1	0.36 ± 0.00 ^a^	0.35 ± 0.00 ^a^	0.33 ± 0.00 ^a^	0.35 ± 0.00 ^a^	0.47 ± 0.00 ^a^	0.42 ± 0.00 ^a^	0.37 ± 0.00 ^a^

Each value is the mean of three measurements ± SD. The mean values with different lowercase letters (a–i) within each column are significantly different (*p* < 0.05).

**Table 3:** Syringe flow test (SFT) and Line spread test (LST) measurements within nectar-thick viscosity ranges (51–350 mPa·s).

Thickener Conc. (%)	Orange Juice		Grape Juice		Apple Juice		Sports Drink		Whole Milk		Low Fat Milk		Soybean Milk	
	SFT (mL)	LST (cm)	SFT (mL)	LST (cm)	SFT (mL)	LST (cm)	SFT (mL)	LST (cm)	SFT (mL)	LST (cm)	SFT (mL)	LST (cm)	SFT (mL)	LST (cm)
0.2	-	-	-	-	-	-	-	-	-	-	-	-	-	-
0.3	-	-	-	-	2.73 ± 0.12 ^h^	9.93 ± 0.21 ^a^	2.93 ± 0.15 ^h^	8.63 ± 0.12 ^a^	4.07 ± 0.12 ^f^	8.80 ± 0.17 ^a^	4.00 ± 0.10 ^f^	7.90 ± 0.17 ^a^	4.13 ± 0.12 ^g^	8.23 ± 0.06 ^a^
0.4	3.77 ± 0.15 ^f^	8.03 ± 0.06 ^a^	1.27 ± 0.12 ^g^	9.4 ± 0.00 ^a^	3.23 ± 0.15 ^g^	8.40 ± 0.10 ^b^	4.20 ± 0.20 ^g^	7.97 ± 0.06 ^b^	5.67 ± 0.23 ^e^	8.10 ± 0.10 ^b^	5.00 ± 0.10 ^e^	7.80 ± 0.10 ^a^	4.87 ± 0.12 ^f^	8.00 ± 0.10 ^ab^
0.5	6.20 ± 0.20 ^e^	7.50 ± 0.00 ^b^	4.23 ± 0.06 ^f^	7.83 ± 0.06 ^b^	5.67 ± 0.06 ^f^	7.63 ± 0.06 ^c^	4.70 ± 0.10 ^f^	7.70 ± 0.10 ^c^	7.50 ± 0.10 ^d^	7.77 ± 0.06 ^c^	7.10 ± 0.17 ^d^	7.33 ± 0.06 ^b^	5.87 ± 0.12 ^e^	7.80 ± 0.26 ^b^
0.6	6.87 ± 0.21 ^d^	7.23 ± 0.15 ^c^	6.17 ± 0.15 ^e^	7.57 ± 0.06 ^c^	6.47 ± 0.06 ^e^	7.37 ± 0.06 ^d^	6.83 ± 0.06 ^e^	7.36 ± 0.06 ^d^	7.97 ± 0.06 ^c^	7.47 ± 0.06 ^d^	7.83 ± 0.21 ^c^	7.03 ± 0.06 ^c^	6.77 ± 0.06 ^d^	6.67 ± 0.06 ^c^
0.7	7.90 ± 0.10 ^c^	6.97 ± 0.06 ^d^	7.17 ± 0.15 ^d^	7.17 ± 0.06 ^d^	7.53 ± 0.06 ^d^	6.90 ± 0.10 ^e^	7.90 ± 0.17 ^d^	6.57 ± 0.06 ^e^	8.47 ± 0.06 ^b^	7.20 ± 0.10 ^e^	8.27 ± 0.12 ^b^	6.77 ± 0.06 ^d^	7.53 ± 0.06 ^c^	6.50 ± 0.10 ^c^
0.8	8.67 ± 0.12 ^b^	6.63 ± 0.06 ^e^	8.33 ± 0.12 ^c^	6.90 ± 0.10 ^e^	8.57 ± 0.06 ^c^	6.57 ± 0.12 ^f^	8.87 ± 0.15 ^c^	6.30 ± 0.05 ^f^	8.77 ± 0.06 ^a^	6.90 ± 0.10 ^f^	8.87 ± 0.12 ^a^	6.47 ± 0.06 ^e^	8.57 ± 0.15 ^b^	6.13 ± 0.21 ^d^
0.9	9.50 ± 0.10 ^a^	6.37 ± 0.06 ^f^	8.67 ± 0.00 ^b^	6.57 ± 0.06 ^f^	9.13 ± 0.12 ^b^	6.20 ± 0.10 ^g^	9.27 ± 0.06 ^b^	6.13 ± 0.06 ^g^	-	-	-	-	9.87 ± 0.06 ^a^	5.73 ± 0.06 ^e^
1	-	-	9.60 ± 0.10 ^a^	6.30 ± 0.10 ^g^	9.77 ± 0.06 ^a^	5.80 ± 0.00 ^h^	9.63 ± 0.06 ^a^	5.86 ± 0.04 ^h^	-	-	-	-	-	-

Each value is the mean of three measurements ± SD. The mean values with different lowercase letters (a–h) within each column are significantly different (*p* < 0.05).

## Data Availability

All the results showed in the manuscript could be requested to the corresponding author who would provide them.
